# How artificial intelligence affects the labour force employment structure from the perspective of industrial structure optimisation

**DOI:** 10.1016/j.heliyon.2024.e26686

**Published:** 2024-02-22

**Authors:** Xiaowen Wang, Mingyue Chen, Nanxu Chen

**Affiliations:** School of Economics, Lanzhou University, Lanzhou, 730000, China

**Keywords:** Artificial intelligence, Industrial structure, Employment structure, Threshold effect, Eight economic regions

## Abstract

To investigate how artificial intelligence (AI) affects the structure of labour force employment, we integrate robotics adoption and employment into this study's model. Based on Chinese provincial panel data from 2010 to 2019, fixed, mediating and threshold effects models and a spatial heterogeneity model were used to empirically test the impact of AI on the employment structure from the perspective of industrial structure optimisation and its mechanisms of action. The findings demonstrate that the impact of AI on the labour force employment structure reflects unique characteristics for China and promotes the advancement of the nation's employment structure. The influence of AI on the labour force employment structure follows a non-linear pattern, fostering labour force employment structure optimisation and upgrading from the perspective of industrial structure optimisation. Further investigation reveals the influence of spatial spillover effects from AI on employment structure optimisation. These research findings have theoretical value and practical significance for optimising China's employment structure in the context of AI.

## 1 Introduction

The rise of the new industrial revolution, characterised by digitalisation, networking and intelligence, has made artificial intelligence (AI) a driving force for the continued advancement of humankind. AI is highly valued by countries worldwide. For instance, Germany's Industry 4.0 aims to create smart manufacturing, Japan's Society 5.0 emphasises the use of AI to serve human beings and the United Kingdom's New Deal for the Artificial Intelligence Sector plans AI development from multiple perspectives to reshape the global industrial division of labour through industrial upgrading and intelligent transformation [1]. The labour force employment structure in the United States and several European countries has begun to polarise as a concomitant phenomenon that is characterised by an increase in high-skilled jobs in low-skilled sectors and a decrease in jobs in medium-skilled sectors [2].

As the foremost emerging economy, China has adopted a national blueprint to seize the transformative prospects presented by the latest industrial revolution wave. In 2016, this blueprint integrated the concept of AI in the 13th Five-Year Plan. Following this, in 2017, the State Council unveiled the New-Generation Artificial Intelligence Development Plan. The report of the 19th National Congress accentuated the fusion of AI with the tangible economy to foster growth. By October 2018, the Political Bureau of the CPC Central Committee convened for its ninth group study, focusing on AI's evolution and future trajectory. The session, chaired by General Secretary Xi Jinping, highlighted the pivotal role of AI as a catalyst for the forthcoming scientific and technological revolution and industrial transformation. General Secretary Xi advocated accelerating the advancement of next-gen AI, pinpointing it as a strategic imperative for China to navigate and leverage the upcoming wave of global scientific, technological and industrial evolution. In February 2020, the Central Committee for Comprehensively Deepening Reform forward proposed to harness digital technologies, including big data, AI and cloud computing, aiming to enhance support for tasks such as epidemic surveillance, virus tracking and management of prevention, control, treatment and logistical allocations during health crises. These initiatives have accelerated technological and industrial change and promoted the deep integration of the real economy with AI. They have also engendered massive investment in advanced equipment embedded with cutting-edge technologies. Owing to the long-standing policy of prioritising capital deepening, coupled with a shortened technology replacement cycle and enterprises' increased labour costs, Chinese companies have replaced human labour with machines to enhance productivity and competitiveness in the new era [3]. Against this background, China's industrial intelligence is entering a phase of rapid development, which is bound to have a profound impact on the nation's employment structure. Notable differences are evident in the industrial intelligence and upgrading process between China's eastern, central and western regions due to unbalanced economic development. The high cost of living in developed regions has a significant impact on the supply and mobility of labour with different skills. Considering the reality of unbalanced regional development, China's labour force employment structure in the new period may have unique characteristics that differ from those of Western industrialised countries [4].

It is widely agreed that AI impacts employment through substitution and creation effects [5]. AI development can lower the cost of machinery and equipment [6], leading firms to replace human labour with robots [7], and increase production automation [8], resulting in substitution effects [4], that ultimately cause job loss [9]. AI development can also have a positive impact on employment. In addition to expanding production scale by reducing costs, promoting capital accumulation and increasing the productivity of machines and equipment [10], AI complements the skills of the labour force in research and development (R&D) and design and communication [11], opening new job opportunities [12]. A lack of consensus remains among scholars regarding how AI affects employment structure, based on the substitution and creation effects [13,14]. In the context of industrial upgrading, research on the impact of AI on employment structure is particularly important for China, where population ageing is becoming increasingly serious. Furthermore, research on the impact of AI on the labour force employment structure tends to adopt a linear perspective, with fewer analyses of non-linear impacts. It is essential to explore non-linear impact trends. Additionally, while the impact of AI on local employment structure is often explored, previous research largely ignores its spatial spillover effects on employment structure.

The contributions of this study are manifold. (1) The study derives inspiration from Bartik's instrumental variables [15], constructing a new regional industrial robot penetration index, using robotics from the International Federation of Robotics (IFR) and Chinese labour employment structural data from 2010 to 2019. We evaluate AI's influence on labour market employment configurations applying instrumental variables techniques, while acknowledging China's unique context. (2) The Development Research Centre of the State Council's Department of Development Strategy and Regional Economy classified mainland China into eight economic territories based on developmental profiles. We employ this regional demarcation to dissect AI's differential effects on employment structure across these zones. (3) This study investigates the non-linear impacts of AI on the labour force employment structure. Analysing the threshold effects, we demonstrate that while AI's impact on employment varies for high and low-skilled labour, with clear threshold effects, such effects are not evident for medium-skilled labour. (4) This study goes beyond the conventional emphasis on industrial structure upgrading, differentiating between advancement and rationalisation of industrial structure to examine the impact of each construct on altering employment structure under AI's mediating role. We posit that the mediating influence of industrial structure rationalisation on employment is comparatively subdued. (5) AI will promote the transformation and upgrading of local industries and drive the upgrading of industries, scale expansion and economic development of surrounding areas, which will generate spatial spillover effects on the employment structure.

## 2 Literature review and hypotheses

### 2.1 Artificial intelligence and employment

The concept of AI was first introduced during the Dartmouth Conference in 1956 [16]. The concept denotes the proficiency of machinery to emulate human actions and execute tasks intelligently, mirroring people's activities [17]. With the advent of the big data era and information technology advancements such as cloud computing and 3D printing, AI technology's evolution has unveiled novel opportunities. AI refers to versatile and practical technology that can be applied to various fields of social production. It is an emerging science and technology that can significantly improve production efficiency. AI technology has potential applications across various domains of social production, representing an emerging scientific field that can advance production efficiency significantly [18]. Scholars continue to contemplate the implications of the rise and rapid development of AI technology on human society.

Previous research regarding the impact of AI on employment structure largely revolves around the two theoretical perspectives of skill-bias theory, which focuses on changes in skill structures, and task-bias theory, which examines task structures [19]. In the early stages of AI, Autor et al. [20] argue, based on skill-bias theory, that AI would indeed impact the labour force employment structure, posing a high risk of job displacement for medium- and low-skilled workers. As automation progresses, Autor et al. [21] observe a polarisation of employment in the labour market, which is characterised by a significant decline in medium-skilled jobs and an emerging U-shaped workforce skill structure. Additionally, Goos and Manning [22] argue that medium-skilled jobs are dominated by procedural tasks with a certain degree of regularity and are subsequently more susceptible to automation. In contrast, low-skilled labour, which is primarily non-procedural and manual, is at a reduced risk of replacement. This gives rise to the phenomenon of employment polarisation. The application of AI is likely to accelerate employment inequality and exacerbate the phenomenon of job polarisation [6].

Some previous findings support the conclusion that AI will lead to a unipolar bias in the structure of human capital demand [23,24]. The fourth industrial revolution will bring significant changes to the labour market's occupational structure. The development of AI technology will increase the demand for knowledge-based and skilled workers, resulting in a pyramid-type occupational skills structure, with the lowest level of low-skilled human capital no longer being viable employment as the bottom of the pyramid is hollowed out [[25], [26], [27]]. Fleming [28] argues that AI has clear organisational boundaries that affect employment. Unemployment is more likely to result from the threat of macro-economic pressure on low-skilled occupations. Trajtenberg [29] suggests that technology can increase human capital accumulation in industries related to healthcare, education and law, particularly for young individuals with good educational backgrounds and technical expertise. However, for labour groups with lower wage rates in replaced industries, the higher cost of human capital reallocation may exacerbate the employment crisis. Bratti and Matteucci [30] conduct an empirical study to investigate the correlation between skill-biased technological advancement and changes in the labour force structure using time-series data. The study demonstrates that technology advancement can substantially boost the demand for skilled labour within firms. Jongwanich et al. [31] examine the influence of technological advancement on labour force employment in economic terms, examining the creation and substitution effects caused by technological progress and the demographic transmission mechanism and philosophical reflections in the non-economic mechanism. The authors explore the particularity of the new round of technological progress led by AI technology, suggesting that AI technology will exacerbate spatial and technological polarisation in the job market. These findings lead to our first hypothesis.

Hypothesis 1 Artificial intelligence development will optimise the workforce employment structure.

### 2.2 Artificial intelligence, industrial structure optimisation and workforce employment structure

Technological progress is found to promote industrial change in two ways. First, the innovation and application of new technology and the integration and development of inherent technology advances the whole industry towards high-quality development, achieving a leap in industrial structure. In other words, the industry can advance to a higher level of technical maturity [32]. Second, technological progress alters the existing demand–supply structure, production resource conditions and factor allocation within the system. This ensures that the industrial structure is effectively matched with technological methods to maximise different production efficiencies [33]. This study investigates the sequence of reasoning through which AI enhances the industrial structure, encompassing its modernisation and rationalisation and concurrently influencing shifts in the employment landscape.

Industrial advancement refers to increased productivity through implementation of automated and digitalised production processes [34]. The use of AI technologies has become widespread in various industries, including healthcare, finance and manufacturing, due to advancements in science and technology which have led to increased efficiency, automation and digitisation in production processes [35]. Application of AI technologies improves production efficiency and enhances industry scalability and innovation, promoting advanced industrial structure. Using the industrial field as an example, AI technology has enabled the automation of multiple production processes through the use of robots, resulting in reduced labour costs and improved production efficiency [36]. AI technology applications have also introduced new innovations into the industrial production process, advancing the industrial structure in the process of intelligent upgrading of production [37]. As the industrial structure evolves, industries that are characterised by low value addition and high labour intensity are expected to progressively diminish. Conversely, industries that are high in value addition, rich in technological intensity and grounded in knowledge are anticipated to advance, culminating in employment structure refinement [38]. Overall, labour mobility will rise, allowing individuals to obtain suitable jobs across various industries.

Hypothesis 2 Artificial intelligence optimises the labour force employment structure through advanced industrialisation.

Industrial structure rationalisation primarily involves the optimisation and coordination of all aspects of the production process [39]. In the past, each link in the production process operated independently, making coordination difficult; however, with the development of AI, a more rationalised industrial structure emerges, resulting in a more efficient production process [40]. In the financial industry, AI has transferred administrative work to machines for processing [41]. Which has improved efficiency and reduced losses caused by human errors [42], leading to optimised processes, reduced costs and increased efficiency. The industry structure has become more rationalised as a result. At the same time, AI has enabled companies to follow market demand more accurately and optimise products and services. This approach, unlike traditional market research, has led to a shorter, increasingly market-responsive and precise product development cycles. As a result, the industrial structure is further rationalised [43]. The relative stability of employment and the balanced development of society can be achieved by rationally allocating resources among different industries. A rationalised industrial structure can balance different decisions for advancing economic development based on productivity, promote resource coordination and rational development among various industries and ultimately optimise the employment structure [44].

Hypothesis 3 Artificial intelligence optimises the labour force employment structure by rationalising the industrial structure.

### 2.3 Non-linear effects of AI on labour force employment structure

As described above, AI advances industrial structure optimisation, blurring production boundaries between sectors and increasing integration and diffusion between AI and the real economy. This integration has had a significant impact on the economy. Based on Metcalfe's law, network spillovers are likely to have a non-linear character. Electronic information products can accelerate the substitution of informatisation in non-information sectors across society, superimposing digital technological capital on these sectors [45]. Therefore, the impact of AI on the employment structure is likely to increase exponentially due to the spillover effects of openness, diffusion, externalities and the extremely low marginal costs of information technology. Previous research demonstrates that AI will have a non-linear impact on total factor productivity [46] and industrial structure upgrading [47]. Additionally, it will have a marginal incremental effect on high-quality development [48] and green economy efficiency [49]. Regarding the non-linear impact of AI on the labour force employment structure, some studies find that it will follow an inverted U-shaped pattern, initially rising before falling [[50], [51], [52]]. AI may impact the employment structure by exhibiting wave recurrence, and its impact on optimising the workforce may have a non-linear threshold effect. The widespread use of AI may significantly increase the demand for highly skilled labour in some areas, while reducing demand for low-skilled labour in others. This shift in the skill mix could cause a non-linear adjustment in the labour market, leaving some groups vulnerable to unemployment while others benefit from expertise in high-skilled areas. Second, AI may alter the industrial structure, resulting in the decline of certain traditional sectors and the emergence of new high-tech industries, which may cause a non-linear migration of labour between industries that could impact the evolution of the employment structure.

Hypothesis 4 The impact of AI on employment within the labour force is non-linear, with a threshold effect resulting from industrial structure optimisation.

To summarise, existing literature examines the nexus between industrial structure and employment, alongside the ramifications of AI evolution on job markets; however, when the concept of industrial structure optimisation is holistically incorporated into analysing the labour market's comprehensive impact, consensus is not yet reached regarding the distinct influences of specific trajectories as the refinement of the industrial framework and advancements in technology, including AI, concurrently bolster one another. Under the innovation-led development paradigm, AI's role in boosting productivity and steering the shift in employment skills is pivotal. Previous research thoroughly examines the repercussions of AI on labour employment, particularly its dual role in job creation and displacement; however, studies seldom dissect AI's role in transforming industrial structure, particularly in discerning between the enhancement and streamlining of these structures and the resultant effects on employment skills. Building on these findings, this study forges a cohesive analytical structure that melds AI progress, industrial structure enhancement and the architecture of employment skills to investigate the influence of AI's advancement on the skills required for employment, mediated through the optimisation of industrial structure, using a mediating effect model and a panel threshold regression model for our analysis.

## 3 Methods

### 3.1 Model setting

Based on hypothesis H1, the following two-way fixed effects benchmark model is designed to systematically analyse the impact of AI on the employment structure.

(1) $$\ln\;Y_{it}=\beta_{0}+\beta_{1}Robot_{it}+\beta_{2}X_{it}+\gamma_{t}+\delta_{i}+\varepsilon_{it}$$

In equation (1), the explanatory variables ln*Y*_*it*_ denote the differently skilled labor force of region *i* in year *t*, respectively. The main explanatory variable Robot_*it*_ is AI development in region *i* in year *t*, and *X*_*it*_ is a set of control variables. In addition, *δ*_*i*_ denotes a region fixed effect, *γ*_*t*_ denotes a time fixed effect, and *ε*_*it*_ is an error term.

The impact of AI on employment structure is multidimensional and may have a phased effect with varying degrees of AI application. This paper aims to explore the potential non-linear relationship between the two and test whether AI will alter the employment structure of the labour force. To achieve this, we draw on the study by Hansen [53] and construct the following threshold regression model (2) based on equation (1):

(2) $$\ln\;Y_{it}=\alpha +\beta_{1}C_{it}+I\left(Is_{it}\leq \lambda_{1}\right)+\beta_{2}C_{it}\times I\left(\lambda_{1}<Is_{it}\leq \lambda_{2}\right)+\beta_{n}C_{it}\times I\left(\lambda_{n-1}<Is_{it}<\lambda_{n}\right)+\beta_{n+1}C_{it}\times I\left(Is_{it}>\lambda_{n}\right)+\theta X_{it}+\delta_{t}+Y_{t}+\varepsilon_{it}$$

The previous theoretical analysis indicates that AI has the potential to alter China's employment structure through advanced and rationalised industrial structures. This paper employs the mediation effect method to investigate the existence of these mechanisms. The econometric model (3) and (4) is constructed as follows:

(3) $$\ln\;M_{it}=\beta_{0}+aRobot_{it}+\beta_{1}X_{it}+\mu_{i}+\delta_{t}+\varepsilon_{it}$$

(4) $$\ln\;Y_{it}=\theta_{0}+c^{\prime }Robot_{it}+bM_{it}+\theta_{1}X_{it}+\mu_{i}+\delta_{t}+\varepsilon_{it}$$

Furthermore, the use of general panel data may result in significant bias in multiple linear regressions. To address this issue, Lesage and Pace [54] proposes incorporating the Durbin model into the standard regression equation (1) and empirically examines the construction of a spatial Durbin model with double fixed effects, as shown in equation (5):

(5) $$\ln\;Y_{it}=\beta_{1}W_{ij}\;\ln\;Y_{it}+\beta_{2}de_{it}+\beta_{3}W_{ij}de_{it}+\beta_{4}X_{it}+\beta_{5}W_{ij}X_{it}+\delta_{i}+\gamma_{t}+\varepsilon_{it}$$

This paper tests two spatial matrices. The first matrix is a geographic distance matrix, introduced using the spatial measurement method, to comprehensively examine the relationship between AI and employment structure. Secondly, the spatial relationship is not accurately reflected by distance alone. When two cities have similar geographical distances, the spillover effect of a highly economically developed city will also impact neighbouring cities, flowing into those with similar economic levels. This may be due to the compatibility of resource allocation and similar economic levels, resulting in an obvious spillover effect. Thus, the spatial economic geography matrix utilises the geographic distance matrix and the economic distance matrix [55]. As shown in equation (6):

(6) $$W_{ij}^{d}=\frac{1}{d_{ij}},\;W_{ij}^{e}=\frac{1}{{\bar{GDP}}_{i}-\bar{GDP_{j}}},\;W_{ij}^{d-e}=\frac{{\bar{GDP}}_{i}-\bar{GDP_{j}}}{d_{ij}}$$

where *d*_*ij*_ is the distance between cities, and ${\bar{GDP}}_{i}$ and ${\bar{GDP}}_{j}$ are the average of the 2011–2020 deflator GDP of province *i* and province *j*, respectively, to obtain the economic geography matrix $W_{ij}^{d-e}$.

## 4 Data

### 4.1 Explained variables

Labour force employment structure (L): The employment structure within the labour force is commonly categorised based on skill structure, as evident in previous literature. Aghion and Howitt [56] argue that technological progress accelerates the life cycle of jobs, causing outdated jobs to disappear and new jobs to be created at an increasingly rapid rate. This results in a significant increase in the frequency of entry and exit of differently skilled labour into and out of the job market. Haltiwanger et al.‘s theory [57] contends that the rapid development of AI technology has decreased the price of new technologies and equipment, prompting enterprises to adopt new technologies and abandon outdated jobs, which further accelerates changes in the proportion of different skills in the employment market.

This study references Sun and Hou [4] to measure the employment structure. This approach involves assessing the proportion of employed individuals across different education levels, which serves as an indicator of their skill level. Specifically, we divide the labour force into low-, medium- and high-skilled categories, classifying individuals with junior high school education or below as low-skilled, those with a high school education as the medium-skilled category and individuals with college education and above as high-skilled.

### 4.2 Explanatory variables

The IFR conducts annual surveys of robot manufacturers worldwide and produces annual country–industry statistics on robotics, which are considered the most authoritative in the world. Since 2006, IFR has supplied annual data on the number of industrial robots active within various industries in China. Acemoglu and Restrepo [2] examine the impact of robotic applications on regional labour markets within the United States through a general equilibrium model, constructing a regional robot penetration index for the United States that is analogous to the Bartik instrument, drawing from the model's insights [15,58]. This study applies this methodology of disaggregating industrial robots at the industry and regional levels. This study matches the industry segments given by China's National Economic Industry Classification (GB/T4754-2017) with those in the IFR data, excluding unspecified industries and data on employed persons and robots that cannot be clearly classified. The resulting dataset provides data on the stock of robots and the number of employed persons in the corresponding industries in the IFR. The measurements used are those given in equation (7):

(7) $$AI_{jht}=\frac{l_{jht_{0}}}{\sum_{j=1}^{n}l_{jht_{0}}}\times \frac{AI_{ht}}{l_{ht_{0}}}$$

where, *j* is the province, *h* is the industry, *t* is the year, *AI*_*jht*_ is the robot penetration rate of *h* industry in province *j* in year *t*, *t*_*0*_ is the base period, $\frac{l_{jht_{0}}}{\sum_{j=1}^{n}l_{jht_{0}}}$ is the share of *h* industry employment in province *j* in the total employment of *h* industry in the whole country in the base period, and $\frac{AI_{ht}}{l_{ht_{0}}}$ is the robot penetration rate of *h* industry in year *t*. The penetration of industrial robots in the industry is broken down to the provincial level using this ratio as the weight. The aim is to investigate the penetration of industrial robots specifically at the province level.

### 4.3 Mediating variables

Industrial structure optimisation: Industrial structure optimisation refers to improving overall economic efficiency, sustainability and competitiveness by rationally adjusting and reconfiguring the weight and structure of various industrial sectors in the economic system. In conjunction with the rapid proliferation of AI in China, significant transformations have taken place in the landscape of domestic industrial structure upgrading. This study aims to enhance the accuracy of depicting the inter-provincial optimisation of industrial structure in China. We achieve this by refining the concept of industrial structure upgrading from a structuralist perspective by including the two dimensions of industrial structure advancement and rationalisation.

(1) Industrial structure advancement (ISA): ISA denotes the vibrant progression in which the industrial structure adheres to the intrinsic rationale of economic expansion, transitioning the industry's value-add from minimal to substantial and advancing the structural tier from elementary to advanced. This progression is frequently propelled by technological advancements and serves as a crucial indicator of the industrial framework's evolution and elevation. Commonly, the complexity of the industrial structure is gauged by the ratio of non-agricultural production to the total industrial output value. However, since China's economy has shifted from being primarily agricultural to industrial and service-based, the proportion of agriculture in overall economic growth has gradually decreased, with the rate of change diminishing each year. Non-agricultural industries, including industrial and service output values, have become increasingly significant, making a simple division between agriculture and non-agriculture imprecise when attempting to capture the evolution of the industrial structure from low to high levels. Therefore, we use the proportion of value added in the tertiary industry to that in the secondary industry to measure ISA.
(2) Industrial structure rationalisation (ISR): ISR refers to the level of coupling between industries. This measure determines whether the development of an industry can effectively drive all aspects of the coordinated operation of resources, achieve optimal resource allocation and reach the goal of balanced development between industries. The Thiel index method is the most commonly used method for this indicator in existing literature on China. The formula for calculating the ISR index is shown in equation (8):

(8) $$TL=\sum_{i=1}^{n}\left(\frac{Y_{i}}{Y}\right)\ln\left(\frac{Y_{i}}{L_{i}}/\frac{Y_{i}}{L}\right)$$

### 4.4 Control variables

We incorporate the following control variables in this study. Urbanisation level (Urb), which is measured by the share of urban population; education investment (Edu), which is measured by the national fiscal expenditure on education in each province; technological innovation expenditure (Tec), which is measured by the share of science and technology in fiscal expenditure; social fixed asset investment (Fix), which is expressed as the logarithm of investment in fixed assets in the society; foreign direct investment, which is measured by foreign direct investment as a percentage of GDP; marketisation level (Mark), which generally reflects the degree of market economy development in a given location, with a higher index indicating a better level of development (it is crucial to note that this index specifically refers to the marketisation index); and financial development level (Df), which is measured by the ratio of year-end deposits and loans to GDP in each province.

### 4.5 Sample and data sources

The study focuses on the 31 provincial administrative regions in China from 2010 to 2019. Provincial data are obtained from the China Statistical Yearbook and the statistical yearbooks of each province in China. Missing data were completed using interpolation. Table 1 presents the descriptive statistics of each variable.

**Table 1:** Descriptive statistics.

	Variable	Obs	Mean	Std. Dev	Min	Max
Explained variables	High	310	2.769	0.447	1.775	4.130
	Mid	310	2.784	0.326	1.306	3.355
	Low	310	4.154	0.244	2.970	4.497
Explanatory variables	Robot	310	3.805	1.900	−4.772	7.235
Intermediate variables	Isa	310	0.044	0.410	−0.694	1.643
	Isr	310	−0.771	0.750	−4.075	0.3443
Control variables	Urb	310	3.998	0.2445	3.121	4.495
	Edu	310	6.401	0.721	4.108	8.074
	Tec	310	4.092	1.131	0.997	7.064
	Fix	310	9.362	0.9155	6.137	10.986
	Fdi	310	−1.402	0.828	−3.050	0.582
	Mark	310	1.971	0.430	−2.137	2.442
	Df	310	0.259	0.359	−0.520	2.762

## 5 Results

We first conduct unit root and cointegration tests for each variable set in the model to verify smoothness and avoid pseudo-regression. Table 2 confirms that each variable passed the unit root test and demonstrates smoothness. However, some variables required first- and second-order differencing, while still being regressed according to the original variables in equation (1). Therefore, a cointegration test is necessary for the panel data model. Table 3 presents the results, indicating that Kao, Pedroni and Westerlund tests are highly significant, passing the cointegration test. The model appears to be relatively smooth, exhibiting no multicollinearity or pseudo-regression phenomena and the regression results are robust.

**Table 2:** Unit root test.

variables	HT test		IPS test	
	statistic	P-value	statistic	P-value
High	0.118	0.000	−4.854	0.000
Mid	−0.281	0.000	−5.869	0.000
Low	0.028	0.000	−4.983	0.000
Robot	0.252	0.027	−4.825	0.000
Urb	0.001**	0.000	−5.639**	0.000
Edu	0.209	0.005	−4.520	0.000
Tec	0.049*	0.000	−4.977*	0.000
Fix	−0.411**	0.000	−2.521*	0.006
Fdi	0.257	0.032	−5.010*	0.000
Mark	0.181*	0.024	−2.061*	0.020
Df	−0.359	0.000	−4.761*	0.000

Note: * and * * denote first-order and second-order differencing of variables.

**Table 3:** Cointegration test.

Check type		High		Mid		Low	
		statistic	P-value	statistic	P-value	statistic	P-value
Kao test	Modified Dickey–Fuller t	2.451	0.007	−4.831	0.000	1.544	0.061
	Dickey–Fuller t	−1.567	0.059	−6.975	0.000	−2.157	0.016
	Augmented Dickey–Fuller t	1.658	0.049	−2.585	0.005	1.935	0.027
Pedroni test	Modified Phillips–Perron t	7.970	0.000	8.153	0.000	8.842	0.000
	Phillips–Perron t	−11.409	0.000	−16.177	0.000	−11.033	0.000
	Augmented Dickey–Fuller t	−10.233	0.000	−10.437	0.000	−13.102	0.000
Westerlund test	Variance ratio	3.858	0.000	3.110	0.001	3.102	0.001

### 5.1 Baseline estimation results

This section empirically tests the impact of AI on China's employment structure using the econometric models presented above. All models control for individual and time factors. Table 4 shows that a one-unit increase in AI significantly contributes to the growth of the high- and medium-skilled labour force by 0.063 and 0.001, respectively. At the 5% level, holding all other variables constant, increasing intelligent technology will result in a 0.063% increase in the high-skilled labour force and a 0.001% increase in the medium-skilled labour force due to the creation effect. However, it will also lead to a 0.001-unit decrease in the growth of the low-skilled labour force. For each one-unit increase in AI, the employment share of lower-skilled workers will decrease by 0.001% due to the replacement effect. This indicates that AI increases the demand for high- and medium-skilled labour while reducing the demand for low-skilled labour. This can be considered an improvement in the labour force employment structure as it indicates upgrading the workforce skill level of the and optimising the occupational structure to meet the needs of economic development and technological change. This can be achieved by improving education, training and motivation for technological innovation. Improving the workforce structure can help society adapt to emerging industries, enhance overall competitiveness and address the employment challenges faced by low-skilled labour. The development of AI technology does not lead to a crowding-out effect on the employment of high- and medium-skilled labour. Instead, it expands the demand for these two types of labour; however, the low-skilled labour force is subject to replacement effects that shrinks with advanced smart technology. The findings confirm Hypothesis 1, which suggests that AI technology has both destructive and creative impacts on different skill groups. These findings align with those of Cong and Yu [59]. At this stage, the development of AI still has the greatest impact on increasing employment opportunities for high-skilled workers. According to our statistical analysis, AI will increase the employment rate of high-skilled workers by 0.063% at a 5% significance level.

**Table 4:** Estimated results.

Variables	High	Mid	Low
Robot	0.063**	0.001**	−0.001*
	(0.026)	(0.000)	(0.000)
Urb	0.391**	1.477***	0.010***
	(0.177)	(0.206)	(0.002)
Edu	0.000**	0.017	0.011
	(0.000)	(0.058)	(0.009)
Tec	0.037*	0.021*	−0.018**
	(0.020)	(0.012)	(0.008)
Fix	−0.067**	−0.000***	0.020**
	(0.025)	(0.000)	(0.008)
Fdi	−0.026**	−0.112***	−0.001
	(0.012)	(0.025)	(0.011)
Mark	−0.168	−0.303***	0.046**
	(0.123)	(0.050)	(0.019)
Df	0.001	0.028	0.038*
	(0.003)	(0.026)	(0.022)
Constant	1.390*	−2.733***	3.513***
	(0.698)	(0.810)	(0.064)
Region FE	Yes	Yes	Yes
Time FE	Yes	Yes	Yes
R^2^	0.8388	0.6978	0.8067
N	310	310	310

In terms of control variables, (1) urbanisation has a significant positive effect on high-, medium- and low-skilled employment. The effect of urbanisation is higher on high- and medium-skilled employment, which may be attributed to the spatial aggregation of physical and talent capital in urban areas. This makes enterprises more interested in hiring high- and medium-skilled labour, which improves the employment structure. At the same time, the refinement of the social division of labour due to population agglomeration also generates more low-skilled jobs, which is consistent with Wu et al. [60]. (2) Investment in education has a positive impact on the employment of individuals with high school and low-level skills; however, it does not have a significant impact on the employment of those with medium- and high-level skills. This is because education investment directly affects literacy, and China has essentially achieved universal 9-year compulsory education. Public attention to education has also significantly risen with increased education investment. This investment in education promotes the transformation of the employment structure from low-to high-skill, aligning with China's national conditions and consistent with the study by Bhorat et al. [61]. (3) Expenditure on science and technology can significantly reduce employment opportunities for low-skilled workers. This is because such expenditure is typically directed towards high-technology industries, which predominantly employ highly skilled workers. As a result, the substitution effect of high-skilled labour can significantly reduce employment for low-skilled workers. This finding is consistent with Liu et al. [62]. (4) Increased social fixed asset investment and marketisation will have a direct impact on China's employment structure and will encourage private and small-to-medium-sized enterprises to participate in intelligent projects and employ low-skilled labour. Additionally, public welfare and new industry jobs are actively cultivated and developed, resulting in a significant increase in the employment of low-skilled and rural surplus labour. (5) Foreign investment is likely to have a negative impact on all three types of skilled labour, with a particularly significant impact on medium- and high-skilled labour. The impact on high-skilled labour is more significant. This is because foreign-invested enterprises generally have advantages in human capital, technology and market development, which increase competition with domestic enterprises in the same industry. This competition has a crowding-out effect on labour employment, especially for medium- and high-skilled labour. This finding is consistent with Chaudhuri and Banerjee [63]. (6) Financial development has a positive influence on labour employment that is only significant for low-skilled labour. This is primarily due to financial development being based on low-cost and low-risk, which reduces enterprise financing constraints, promotes enterprise technological innovation and scale expansion, generates external economies of scale, creates jobs for more labour and advances human capital agglomeration. Notably, this impact has a lag effect; thus, its impact on labour employment must be verified.

### 5.2 Endogeneity test and robustness tests

#### 5.2.1 Endogeneity test

We first employ the instrumental variable method. To ensure that the instrumental variable is correlated with the endogenous explanatory variables and independent of the random disturbance term, we reference the method proposed by Sun and Hou [64] selecting the density of long-distance optical fibre cables in each province as the instrumental variable. The estimation is conducted using the two-stage least squares (2SLS) method. The correlation between the density of long-distance fibre optic cables and AI in each region is significant because fibre optic sensors in long-distance fibre optic cables are basic key components of industrial robots. Additionally, the construction of long-distance fibre optic cables is exogenously determined and is not affected by the employment structure. Hence, it is appropriate to use long-distance optical cable as an instrumental variable. Long-distance cable density is defined as the length of long-distance optical cable per square kilometre in each province. The estimation results are presented in the first three columns of Table 5, indicating that a one-unit increase in AI results in a 0.376% increase in high-skilled employment and a 0.397% increase in medium-skilled employment, while low-skilled employment decreases by 0.003%. The 2SLS regression results do not significantly differ from our baseline regression, except for the size of the coefficients, which is consistent with the previously estimated results.

**Table 5:** Endogeneity test.

Variables	High	Mid	Low	High	Mid	Low
Robot	0.376**	0.397***	−0.003***	0.082***	0.003***	−0.005***
	(0.157)	(0.139)	(0.001)	(0.029)	(0.001)	(0.002)
cons	1.787	4.462***	5.961***	2.307***	−10.598	12.072
	(3.105)	(2.817)	(0.494)	(0.362)	(10.047)	(22.490)
Control	Yes	Yes	Yes	Yes	Yes	Yes
Region FE	Yes	Yes	Yes	Yes	Yes	Yes
Time FE	Yes	Yes	Yes	Yes	Yes	Yes
N	310	310	310	310	310	310

To address the issue of insufficient endogeneity control caused by omitted variables, the economic development level (Eco), the cost of living (Liv) and the innovation environment (Inn) are introduced into the model and the specific estimation results are presented in the last three columns of Table 5.

#### 5.2.2 Robustness tests

We first replace the explanatory variables. Similar to the endogeneity test, By comparing the educational attainment of the employed and the proportion of persons with a tertiary education or above in the broad industry categories of the China Yearbook of Labour Statistics, the employment demand for low-skilled, medium-skilled and high-skilled labour is measured by the difference between the average wage growth of the employed in agriculture, forestry, fishing and animal husbandry, mining and manufacturing and the average wage growth of all employed in the provinces. The results of the estimation are presented in columns (1)–(3) of Table 6. We also revise the model estimation methodology. To account for the typical inertia of macro-economic variables, model (1) includes first-order lagged terms of high-, medium- and low-skilled employment. This model is extended to a dynamic panel model, which was estimated using a systematic generalised method of moments (GMM). The system GMM estimation results are presented in Table 6, columns (4)–(6). The results remain robust, indicating that the coefficient for AI significantly increases employment for medium- and high-skilled workers while decreasing employment for low-skilled workers at a 1% significance level.

**Table 6:** Robustness tests.

variables	(1)	(2)	(3)	(4)	(5)	(6)
	High	Mid	Low	High	Mid	Low
Robot	1.509***	0.002***	−0.003***	0.266***	0.253**	−0.141***
	(0.385)	(0.000)	(0.000)	(0.101)	(0.123)	(0.046)
AR (1)				0.002	0.038	0.024
AR (2)				0.146	0.474	0.261
Sargan-test				0.875	0.464	0.532
cons	−14.563***	−17.056**	5.379	5.964***	−0.917	−0.783
	(2.666)	(6.868)	(3.575)	(1.718)	(1.753)	(1.055)
Control	Yes	Yes	Yes	Yes	Yes	Yes

### 5.3 Heterogeneity analysis

#### 5.3.1 Regional variations

We next analyse the impact of varying degrees of AI development on employment structures across different regions. Previously, the division of China's three major economic regions into east, central and west was relatively crude and did not facilitate an in-depth analysis of regional differences. The Development Research Centre of the State Council divides the nation into eight comprehensive economic zones based on provinces' spatial proximity, the degree of proximity of the resource endowment, level of economic development and social structure. This approach is more comprehensive for examining regional issues. This study examines regional variations in the impact of AI on employment structures across the eight comprehensive economic zones.

Table 7 presents the estimation results, indicating that AI has a positive impact on the employment of high-skilled labour in all eight regions. In the northeast and middle Yellow River regions, AI development significantly reduces the demand for medium-skilled labour while increasing the demand for high- and low-skilled labour, exhibiting employment polarisation trends.

**Table 7:** Heterogeneity analysis.

Variables	High	Mid	Low
Northern Coastal Economic Zone	0.028***	1.934**	−0.807***
	(0.036)	(0.400)	(0.103)
Eastern Coastal Economic Zone	0.237**	0.012**	−0.424**
	(0.032)	(0.002)	(0.063)
Southern Coastal Economic Zone	0.568*	0.228*	−0.226*
	(0.174)	(0.064)	(0.066)
Northeast Economic Zone	0.211***	−0.001**	0.0242**
	(0.018)	(0.000)	(0.0040)
Mid-Yellow River Economic Zone	0.459***	−0.045***	0.0543**
	(0.077)	(0.007)	(0.0094)
Middle Yangtze River Economic Zone	0.546	0.621***	−0.239**
	(0.300)	(0.098)	(0.049)
Southwest Economic Zone	0.426***	0.010*	−0.001***
	(0.098)	(0 0.004)	(0.000)
Northwest Economic Zone	0.454*	0.357**	−0.147*
	(0.1497)	(0 0.083)	(0.047)

Upon further observation, the effect of AI on highly skilled labour in the middle reaches of the Yellow River is greater than that in the northeast, resulting in a 0.2476% increase in the employment ratio of highly skilled labour compared with that in the northeast. In the process of new industrialisation, the middle reaches of the Yellow River region has experienced a significant increase in demand for high-skilled talent due to the vigorous development of pillar industries such as modern chemical, pharmaceuticals, new materials and new energy automobile industries. This demand is the result of regional growth and expansion in these industries. The growth in inputs to the high-level segments of industrial value chains has made it a base for the advanced equipment manufacturing industry. The demand for workers engaged in the maintenance of raw materials and finished products, as well as other basic work, is also a focus. Therefore, the role of AI in promoting the employment of low-skilled labour is relatively significant.

The development of AI in the eight regions also has a positive impact on the employment of medium- and high-skilled workers. The northern, eastern and southern economic regions are considered the three representative regions for the development of smart manufacturing. Unlike the developed countries in the West, these regions do not exhibit employment polarisation. Instead, they show the characteristics of unipolar polarisation. The optimisation of employment structure is most significant in the northern and coastal economic regions, which is consistent with Sun and Hou [4]. This is because the three regions are actively transitioning from labour-intensive industries to knowledge- and capital-intensive industries, exhibiting a high skill bias, which further supports the findings of this study. The contemporary era of socio-economic development has witnessed an increasing trend towards specialisation and a greater concentration of highly skilled workers in highly skilled sectors. As the coastal region moves towards becoming a scientific and technological innovation centre with global influence, it has collaborated with numerous colleges and universities to establish AI research institutes, develop core support software and control systems for intelligent manufacturing and create an industrial internet manufacturing resource platform. This will inevitably increase the demand for a high-skilled workforce. The workforce should not only possess general, professional and technical knowledge but also internet literacy, innovation skills and a commitment to lifelong learning, which is essential to adapt to the rapidly changing technological landscape in the age of AI.

Table 7 shows that AI development in the Southern Coastal Economic Zone has the greatest positive impact on the highly skilled labour force. At the 1% significance level, for every 1% increase in AI, the employment of the highly skilled labour force in the Zone increases by 0.568. The percentage increase of 568% is primarily attributed to the rapid economic development of this zone in recent years. The industrial sector has stabilised with a focus on highly processed manufacturing industries, as the late stage of heavy industrialisation is prioritised. The pattern of technology-intensive industries centred on electronic communication equipment manufacturing remains unchanged. The labour force continues to shift from traditional to modern sectors, moving towards higher labour productivity industries and gradually upgrading to capital- and technology-intensive industries, which require a more highly skilled workforce. This aligns with Fu [65]. At the 1% significance level, the Northeast Economic Zone shows the smallest demand for high-skilled labour, indicating that for every 1% increase in AI, the employment of high-skilled labour in this Zone only increases by 0.2110%. This is primarily attributable to the region's recent economic downturn and the dominance of heavy industry in its industrial base. As the industrial structure ages and regional advantages weaken, the capital investment for industrial development remains large. The structural adjustment of the northeastern industrial base is facing a dilemma of a heavy and large ship that is not easy to turn around due to the considerable number of medium- and large-sized enterprises. This makes it difficult to attract highly skilled labour for employment.

#### 5.3.2 Temporal variations

The State Council released the New-Generation Artificial Intelligence Development Plan on July 20, 2017. The plan establishes China's AI development goals for 2020, 2025 and 2030, proposing a series of specific policy measures to promote continuous innovation and AI technology. The focus includes increasing research efforts, promoting industrial development, strengthening training and talent team building and improving safety, security and legal systems. As shown in Table 8, before the plan's implementation in 2017, AI had a limited role in reducing low-skilled employment and a relatively weak role in promoting high- and medium-skilled employment; however, after implementation in 2017, AI significantly increased the employment of high- and medium-skilled labourers while reducing the employment of low-skilled labourers. The plan's implementation has led to an increase in AI R&D in various regions, resulting in a higher demand for advanced technical, data science and engineering skills. This has also led to an increase in demand for high- and medium-skilled jobs; however, some low-skilled jobs that involve repetitive tasks and simple operations are more easily replaced by automation technology, and the application of AI may lead to a reduction in the demand for these jobs.

**Table 8:** Temporal variations.

Year	Year<2017			Year ≥ 2017		
Variables	High	Mid	Low	High	Mid	Low
Robot	0.008*	0.009***	−0.011**	0.142**	0.462**	−0.130***
	(0.005)	(0.003)	(0.004)	(0.029)	(0.220)	(0.043)
cons	39.959***	−6.962	49.810***	−11.773*	−0.818	4.385**
	(8.067)	(8.872)	(15.188)	(3.863)	(1.689)	(1.959)
Control	Yes	Yes	Yes	Yes	Yes	Yes
Region FE	Yes	Yes	Yes	Yes	Yes	Yes
Time FE	Yes	Yes	Yes	Yes	Yes	Yes
N	310	310	310	310	310	310

### 5.4 Threshold effects and mechanism tests

#### 5.4.1 Threshold effects

Is there a threshold for industrial structure, and does AI have varying effects on employment structure at different levels? This study next considers the two previously introduced threshold variables of advanced industrial structure and ISR using a panel threshold model to estimate and explain the employment effects of AI. First, we conduct a test of the threshold effect. Table 9 applies a single threshold test for high- and low-skilled labour on industrial structure improvement, with a threshold value of 1.4055 and 1.4131, respectively. No threshold effect is evident for medium-skilled employment.

**Table 9:** Threshold existence, estimates and confidence interval tests.

Workforce	Threshold model	Threshold value	F-value	P-value	Critical values for different significance levels		
					1%	5%	10%
High	Single Threshold	1.4055	84.40	0.000	19.0784	21.8540	28.1302
Low	Single Threshold	1.4131	25.40	0.020	18.4333	21.6364	26.1367

Table 10 shows that when ISA of the provincial region is below 1.4055, a 1% increase in AI results in a 0.011% increase in high-skilled employment. When the advanced industrial structure is above this threshold, this promotion of high-skilled employment is further increased to 0.034%. In industries with lower complexity, the impact of AI on high-skilled employment may be less significant due to low-skilled industries being relatively straightforward and requiring less high-skilled labour. In contrast, AI has a more substantial impact on high-skilled employment in industries with higher complexity because industries with high levels of technological content require a greater amount of skilled, high-level labour.

**Table 10:** Threshold model.

Variables	High	Low
Robot≤1.4055	0.011***	
	(0.002)	
Robot˃1.4055	0.034***	
	(0.004)	
Robot≤1.4131		−0.010*
		(0.005)
Robot˃1.4131		−0.288***
		(0.007)
cons	−37.043***	179.727***
	(7.035)	(48.044)
Control	Yes	Yes
R^2^	0.7568	0.6780

When ISA falls below the threshold value of 1.4131, every 1% increase in AI development decreases low-skilled employment by 0.010%. This disincentive to low-skilled employment increases to 0.288% when the industrial structure is above this threshold. At lower levels of industrial structure, there may be more traditional, labour-intensive industries with a higher demand for low-skilled labour. As AI development progresses, it may lead to automation and technological substitution that reduce the demand for low-skilled labour, resulting in a decrease in low-skilled employment.

Table 11 tests ISR against a single threshold for high-skilled labour, with a threshold value of 3.2981. No threshold effect is evident for medium-skilled employment. Low-skilled employment reveals a double threshold effect, with a single threshold value of 2.0999 and a double threshold value of 3.2401.

**Table 11:** Threshold existence, estimates and confidence interval tests.

Workforce	Threshold model	Threshold value	F-value	P-value	Critical values for different significance levels		
					1%	5%	10%
High	Single Threshold	3.298	33.77	0.020	21.5623	26.4386	37.1186
Low	Single Threshold	2.100	12.90	0.073	11.2524	16.5818	28.9362
	Double Threshold	3.240	19.57	0.070	17.9203	21.1132	28.3158

Table 12 shows that if ISR is below the threshold value of 3.2981, a 1% increase in AI results in a 0.007% increase in high-skilled employment; however, if the rationalisation of industrial structure exceeds the threshold value, AI leads to a significant increase in high-skilled employment, with a 0.018% rise for every 1% increase in the level of AI development. When the rationalisation of the industrial structure is below the threshold value of 3.2981, this may indicate a more chaotic, uncoordinated or overly competitive circumstances in the industry. In such cases, the development of AI may have a small boosting effect on high-skilled employment. Ineffective skill matching and resource allocation in a chaotic industrial structure may be the reason that the development of AI can only promote limited growth in high-skilled employment. A more rational and orderly industrial structure, indicating a higher degree of specialisation, skill matching and industrial synergy may emerge once the industrial structure is rationalised beyond a certain threshold. In this case, AI development provides a significant boost to high-skilled employment. The rationalisation of industrial structure may make it easier for high-skilled labour to integrate into innovation- and technology-intensive industries, benefitting more from the development of AI.

**Table 12:** Threshold model.

Variables	High	Low
Robot≤3.2981	0.007***	
	(0.003)	
Robot˃3.2981	0.018***	
	(0.007)	
Robot≤2.0999		−0.070***
		(0.013)
2.0999˂Robot≤3.2401		−0.079***
		(0.013)
Robot≥3.2401		−0.094***
		(0.012)
cons	−21.207***	4.275***
	(6.603)	(0.150)
Control	Yes	Yes
R^2^	0.691	0.745

When ISR is below 2.0999, every 1% increase in the level of AI development reduces low-skilled employment by 0.070%. This may be because in the case of an insufficiently rationalised industrial structure, the application of AI is more likely to replace low-skilled labour, leading to a decline in employment. When ISR is between 2.0999 and 3.2401, the suppressive effect on low-skilled employment rises to 0. At the 0.79% stage, the impact of AI on low-skilled employment begins to increase gradually as the level of ISR rises. This may be because ISR places low-skilled labour under greater substitution pressure in more complex and technology-intensive environments. After ISR crosses the second threshold of 3.2401, this disincentive effect further increases to 0.094%.

#### 5.4.2 Mechanism tests

To investigate the impact of AI on employment structure in China, this study analyses the mechanism of industrial structure optimisation affected by AI. Table 13 presents the regression results. To avoid single-indicator evaluation errors, this study comprehensively examines the mechanism of industrial structure optimisation. We next introduce the ISA and ISR intermediary indicators to examine the role of industrial structure in depth [66].

**Table 13:** Mechanism test.

Variables	(1)	(2)	(3)	(4)	(5)	(6)	(7)	(8)
	Isa	High	Mid	Low	Isr	High	Mid	Low
Robot	0.262***	0.083***	0.003***	−0.001**	0.013*	0.003**	0.002**	−0.005**
	(0.025)	(0.029)	(0.001)	(0.001)	(0.001)	(0.001)	(0.001)	(0.002)
Isa		0.157***	1.892**	−0.041*				
		(0.040)	(0.714)	(0.020)				
Isr						0.073***	0.026**	−1.426*
						(0.018)	(0.010)	(0.8*0*3)
cons	3.082***	1.800***	−7.912	3.692***	23.425	35.374***	−44.612***	68.879***
	(1.045)	(0.224)	(5.023)	(0.074)	(22.836)	(2.712)	(11.745)	(5.377)
Control	Yes	Yes	Yes	Yes	Yes	Yes	Yes	Yes
Region FE	Yes	Yes	Yes	Yes	Yes	Yes	Yes	Yes
Time FE	Yes	Yes	Yes	Yes	Yes	Yes	Yes	Yes
R^2^	0.788	0.842	0.647	0.801	0.372	0.834	0.651	0.836

Table 13 shows that both ISA and ISR can act as intermediaries in the process of AI affecting employment structure. The intermediary effect of ISA is stronger, enhancing the employment of medium- and high-skilled labour due to the emergence of various intelligent technologies, new processes and business models, which change the demand for human resources to advance high-end, diversified and compound development. New intelligent technologies and business models emerge with the advancement of science and technology, resulting in a significant shift in the demand for human resources towards increased demand for medium- and high-skilled talent. This verifies Hypotheses 2 and 3. In comparison, the mediating role of ISR is relatively weak. This is because, unlike industrial advancement which pursues industrial upgrading, industrial rationalisation focuses on coordination between industries and the rationality of structural distribution. Two current circumstances may lead to the influence of ISR being less obvious. One is that the current rational adjustment between industrial structure is not as efficient and smooth as expected. The impact of AI on industrial structure is primarily seen in industrial upgrading, while the impact on ISR may have a time lag and could be in the critical period of transformation from quantitative to qualitative change.

### 5.5 Further study: spatial spillover effect

We next employ Moran's index (Moran's I) as the test metric to evaluate the significance of AI's spatial spillover. As shown in Table 14, the global Moran's I registers above zero in both scenarios involving distinct spatial weight matrix configurations. For AI and the trio of employment structures, the null hypothesis positing a lack of spatial interdependence is decisively refuted with a significance level of 1%. This indicates that AI and low-, medium- and high-skilled labour force employment structure is characterised by spatial agglomeration and spatial dependence.

**Table 14:** Moran Index,2010–2019.

Year	AI		High		Mid		Low	
	Moran's I	P	Moran's I	P	Moran's I	P	Moran's I	P
2010	0.051***	0.005	0.074***	0.000	0.106***	0.000	0.099***	0.000
2011	0.053***	0.004	0.072***	0.000	0.063***	0.003	0.082***	0.000
2012	0.054***	0.004	0.080***	0.000	0.065***	0.002	0.089***	0.000
2013	0.053***	0.005	0.096***	0.000	0.057***	0.005	0.088***	0.000
2014	0.050***	0.006	0.095***	0.000	0.036***	0.024	0.082***	0.000
2015	0.049***	0.006	0.082***	0.000	0.072***	0.001	0.101***	0.000
2016	0.048***	0.007	0.096***	0.000	0.065***	0.002	0.108***	0.000
2017	0.046***	0.008	0.094***	0.000	0.057***	0.004	0.104***	0.000
2018	0.045***	0.009	0.107***	0.000	0.056***	0.004	0.116***	0.000
2019	0.043***	0.009	0.091***	0.000	0.031***	0.028	0.090***	0.000

Table 15 shows the results of the spatial econometric regression based on two different spatial weights. The findings indicate that AI increases the level of medium- and high-skilled employment while reducing the level of low-skilled employment. This is primarily because AI promotes the emergence of new technologies and industries and emerging industries are typically more technology-intensive and require higher levels of expertise and skills to perform the relevant jobs. Therefore, the proliferation of AI provides more opportunities for medium- and high-skilled employment. Furthermore, AI and machine learning technologies can be applied to repetitive and mechanical tasks, which are typically low-skilled jobs. Automating these tasks can increase productivity and efficiency, while reducing the need for human resources and decreasing the demand for low-skilled jobs.

**Table 15:** SDM

Variables	W1			W2		
	High	Mid	Low	High	Mid	Low
Robot	0.001**	0.003**	−0.006*	0.001*	0.003**	−0.005*
	(0.000)	(0.002)	(0.003)	(0.000)	(0.002)	(0.003)
W*Robot	0.002***	0.016*	−0.034**	0.002***	0.026***	−0.036**
	(0.000)	(0.009)	(0.016)	(0.001)	(0.008)	(0.014)
Control	Yes	Yes	Yes	Yes	Yes	Yes
Region FE	Yes	Yes	Yes	Yes	Yes	Yes
Time FE	Yes	Yes	Yes	Yes	Yes	Yes
R^2^	0.8028	0.5080	0.7956	0.7990	0.5011	0.7913

Table 16 shows that AI has a positive effect on medium- and high-skilled employment at the 1% level, while having a negative effect on low-skilled employment at the same level, under the two spatial weighting matrices. This suggests that AI is effective in enhancing medium- and high-skilled employment and reduces low-skilled employment. Specifically, positive effects on medium- and high-skilled employment and a negative effect on low-skilled employment are found, as shown by the two spatial weighting matrices at the 1% level. Second, AI in a region has a significant impact on employment. Notably, these findings further validate the benchmark regression. Finally, the two spatial weighting matrices demonstrate that medium- and high-skilled employment have a positive indirect effect, while low-skilled employment has a significantly negative indirect effect. This indicates that AI has a modifying effect on the employment structure with a significant spillover effect between regions, causing the modification of the employment structure in other regions.

**Table 16:** Space Overflow.

Variables	W1			W2		
	High	Mid	Low	High	Mid	Low
Direct effect	0.001***	0.005**	−0.007**	0.001**	0.006***	−0.007**
	(0.000)	(0.002)	(0.003)	(0.000)	(0.002)	(0.004)
Indirect effects	0.004***	0.057*	−0.075**	0.004***	0.079***	−0.079**
	(0.001)	(0.032)	(0.037)	(0.002)	(0.029)	(0.037)
Total effect	0.004***	0.061*	−0.082**	0.005***	0.085***	−0.086**
	(0.002)	(0.034)	(0.040)	(0.002)	(0.031)	(0.039)
Control	Yes	Yes	Yes	Yes	Yes	Yes
Region FE	Yes	Yes	Yes	Yes	Yes	Yes
Time FE	Yes	Yes	Yes	Yes	Yes	Yes

## 6 Discussion, conclusions and policy implications

### 6.1 Discussion

This study's findings offer valuable insights into the effects of AI on China's labour force employment structure. It is important to compare these findings with existing research in this area.

(1) The study revealed that AI's impact on the employment structure of the labour force reflects China's uniqueness as it drives the employment structure of medium- and high-skilled labour. This is consistent with existing research [67,68] that shows AI technologies tend to replace routine and repetitive tasks, leading to a shift in labour demand towards higher-skilled jobs.
(2) The study emphasises the non-linear relationship between AI and labour force employment structure. When AI surpasses a certain threshold of industrial structure optimisation, its impact on employment of high- and low-skilled labour becomes more pronounced. This finding supports the perspective that AI technologies are more likely to enhance rather than entirely replace highly skilled workers [69,70].
(3) The study identifies two indirect mechanisms of the impact of AI on the employment structure, industrial structural upgrading and rationalisation. These findings contribute to understanding the complex channels through which AI affects the labour market and add to the existing literature that examines the relationship between AI adoption and industrial restructuring.
(4) The study also shows that AI has spatial spillover effects on optimising employment structure, indicating that the impact of AI on labour force employment structure extends beyond individual regions and into neighbouring areas. This result is consistent with the argument that AI adoption has knock-on effects and can shape the labour market on a wider geographical scale [71,72]. Comparing this finding with existing research on AI spatial spillovers and employment structure provides additional insights into the geographic dynamics of AI's impact.

### 6.2 Conclusions

This paper examines the impact of AI on the employment structure of the labour force during industrial structure upgrading in China from 2010 to 2019. The study uses panel data of provincial robots and labour force employment and employs the fixed effect model, mediating effect model, threshold effect model, and spatial econometric model. The findings indicate that:

(1) The impact of AI on China's employment structure is specific to China and promotes a shift towards a more advanced labour force. This is achieved by replacing low-skilled workers with AI while increasing the employment of middle- and high-skilled workers. This result remains significant even after endogeneity and robustness tests.
(2) Upon analysing the results of regional heterogeneity, it was found that most of the eight economic zones in China exhibit varying degrees of unipolar characteristics in their employment structure with the addition of AI. Additionally, examining variations over time revealed that the plan's execution resulted in a marked rise in the employment figures for labor with medium and high skill levels, whereas there was a concurrent decrease in the employment of workers with low skill sets.
(3) Threshold effect analysis suggests a non-linear relationship in how AI influences the employment patterns of highly skilled versus low-skilled workers. This conclusion is drawn from empirical data rather than subjective judgment. As AI development surpasses specified levels of ISA and ISR, the influence on the employment configuration of skilled workers intensifies, revealing a pronounced optimisation of the labour force structure.
(4) AI exerts not merely a direct influence but also facilitates the refinement and enhancement of China's employment architecture within the labor force via two ancillary channels: ISA and ISR. Of these, ISA serves as a more potent mediating factor, bolstering the employment of medium- and high-skilled labor.
(5) The spatial Durbin model indicates a spatial spillover presence of AI. Furthermore, it demonstrates that through this spillover effect, AI is capable of optimising the labor force's employment configuration. Emphasizing the importance, optimising the structure of labor force employment is crucial.

### 6.3 Policy implications

Based on the research conclusions above, this paper proposes the following countermeasures:

(1) Strengthen the construction of intelligent talent pools to support high-quality human resources. Professional and technical talent is a core element for promoting the development of industrial intelligence. Cultivating high-skilled talent should be an important task of contemporary higher education. To achieve this, policymakers should increase investment in education and provide financial support for intelligent talent development in scientific research institutions. It is also crucial to encourage colleges and universities to establish relevant specialties that align with the nation's development strategy and goals of industrial intelligence, such as intelligent manufacturing, AI and big data analysis. This will cultivate high-quality talent with comprehensive interdisciplinary capabilities, further strengthening the construction of intelligent talent.
(2) Develop AI strategies that are tailored to local conditions to avoid causing unemployment panic and job market imbalances through indiscriminate implementation of large-scale machine replacement. The southern coastal areas can promote the planning and layout of an AI industrial chain to become a model for the development of China's AI and establishing AI industry clusters. Meanwhile, the northeastern region should increase capital investment and efforts to accelerate the application of AI technology on the ground and expand the scope of AI pilots.
(3) Optimising the industrial structure and promoting industrial intelligence are crucial for China's economy. Our threshold effect analysis demonstrates that the level of industrial intelligence has varying impacts on the employment structure under different industrial structures. Due to continuous advancement in intelligence capabilities, refining the industrial framework and endorsing industrial intelligence are pivotal for China's economic evolution and enhancement. The focal point of present global economic rivalry is embodied in the realm of AI. There is a pressing need to accelerate the growth of clusters within the AI industry, foster innovation in intelligent technology and deliver technical reinforcement for industrial intelligence enhancement. Prioritising the smart transformation of conventional sectors has expedited the transition of the industrial composition from basic, low-added-value processing to sophisticated, high-value-added manufacturing, which has facilitated the intelligent transformation of primary, secondary and tertiary industries, resulting in improved production efficiency and reduced production costs. Accelerating the transformation and upgrading of traditional industries can address the existing difficulties in finding employment and recruiting suitable employees and imbalances in matching the demand for skills. Additionally, fully leveraging the enormous potential of AI to optimise the employment structure can further contribute to this solution.
(4) Promote the development of AI and build a multi-centre development pattern of AI. AI can optimise employment structure in peripheral regions through spatial spillover effects. To fully use the spatial spillover effect of AI, it is essential to leverage China's vast market, boost R&D investment, encourage the exploration of AI core technologies, develop new AI products and technologies, promote the widespread adoption of AI technology and equipment and plan for the construction of an AI development centre area to radiate and drive the adoption of AI in peripheral areas. This will promote the development of new industries and modes of operation and enhance regional employment by fully leveraging the spatial spillover effects of AI development.

Although this study has significant theoretical and practical importance, it does have some limitations that cannot be ignored. First, the study only focuses on the circumstances in China and does not consider other countries; therefore, differing nations' attributes may lead to lower generalisability of the results. Second, this paper does not fully consider other factors that may affect the employment structure of the labour force such as trade friction, policy changes or even the ongoing COVID-19 pandemic. To address the research limitations of this study, future research could take a cross-country comparative approach to examine the impact of AI application on the labour markets of different countries. This study's results could be validated for generalisability, providing a more comprehensive understanding of AI's impact on the global labour force employment structure. Additionally, future research could consider introducing other factors that may affect employment structure such as trade friction, policy changes, epidemics and other shocks. Such considerations can enhance the comprehensiveness of the research, accurately reflect the actual circumstances and provide a better explanation of the complexity of changes in employment structure. In addition, future studies could employ a longitudinal research design to track and analyse the long-term impact of the gradual spread of AI on the labour force structure to understand the trajectory of this trend more comprehensively.

## Data availability statement

The datasets generated and analyzed during the current study are available from the corresponding author on reasonable request.

## Additional information

No additional information is available for this paper.

## Funding

Funding Study on the Construction of the Economic Belt in the Hexi Corridor(2022ZD009).

## CRediT authorship contribution statement

**Xiaowen Wang:** Supervision, Investigation. **Mingyue Chen:** Writing – original draft, Visualization, Software, Data curation. **Nanxu Chen:** Writing – review & editing, Supervision.

## Declaration of competing interest

The authors declare that they have no known competing financial interests or personal relationships that could have appeared to influence the work reported in this paper.
